# The effect of body size and inbreeding on cancer mortality in breeds of the domestic dog: a test of the multi-stage model of carcinogenesis

**DOI:** 10.1098/rsos.231356

**Published:** 2024-01-31

**Authors:** Leonard Nunney

**Affiliations:** Department of Evolution, Ecology, and Organismal Biology, University of California Riverside, 900 University Avenue, Riverside, CA 92521, USA

**Keywords:** cancer, multi-stage model, dog breeds, inbreeding, lifespan, metabolic rate

## Abstract

Cancer is a leading cause of death in domestic dogs. Deaths due to cancer vary widely among breeds, providing an opportunity for testing the multi-stage model of carcinogenesis. This model underpins evolutionary and basic studies of cancer suppression and predicts a linear increase in cancer with breed size, an expectation complicated by bigger breeds having a shorter lifespan (decreasing risk). Using three independent datasets, the weight and lifespan of breeds provided a good fit of lifetime cancer mortality to the multi-stage model, the fit suggesting many canine cancers are initiated by four driver mutations. Of 85 breeds in more than one dataset, only flat-coated retriever showed significantly elevated cancer mortality, with Scottish terrier, Bernese mountain dog and bullmastiff also showing notable risk (greater than 50% over expected). Analysis of breed clades suggested terriers experience elevated cancer mortality. There was no evidence that the lower mass-specific metabolic rate of larger breeds reduced cancer risk. Residuals indicated increased breed inbreeding shortened expected lifespan, but had no overall effect on cancer mortality. The results provide a baseline for identifying increased breed risk for specific cancers and demonstrate that, unless selection promotes increased cancer suppression, the evolution of larger longer-lived animals leads to a predictable increased cancer risk.

## 1. Introduction

The domestic dog (*Canis lupus familiaris*) is not typically considered to be a ‘model organism’; however, it exhibits more phenotypic diversity than any mammalian species [1]. This diversity makes dogs ideal for investigating the effects of variation in a wide range of characteristics including longevity and the causes of mortality [1–5]. The value of the dog for such investigations is further enhanced by the existence of veterinary and breed-specific databases that document many of these patterns (e.g. the Veterinary Medical DataBase (VMDB), http://www.vmdb.org).

It has been suggested for some time that cancer research is one area where domestic dogs can provide valuable insight [6–16]. Dog breeds differ substantially in their risk of dying from cancer [4,17,18], ranging in a US dataset from 4% (Miniature Pinscher) to 55% (Bernese Mountain Dog) [18], and different breeds often differ in the types of cancer to which they are most susceptible [14–16,19,20]. As a result, studies of canine cancer are becoming increasingly important in the development of anti-oncogenic drugs [14,21].

Cancer is typically considered to be a mutation-driven process resulting from the accumulation of a set of inherited and/or somatic ‘driver’ mutations in an individual cell, a phenomenon referred to as multi-stage carcinogenesis. This model was first proposed 70 years ago [22], and, assuming a given cancer is rare, the simplest form of the multi-stage model predicts that its prevalence (= *p*) by age *T* is defined by

1.1 $$p=C{\left(ukT\right)}^{M},$$

where *C* is the number of at-risk cells, *k* is their rate of cell division, *u* is the somatic mutation rate for driver mutations and *M* is the number of driver mutations required to initiate cancer [23].

An important prediction of equation (1.1) is that, all else being equal, the incidence of cancer in different animals (or tissues) will increase with cell number (*C*) and the number of times each cell divides (*kT*). As a result, bigger long-lived animals are expected to have a greater lifetime risk of cancer [24], a result that follows from the simple expectation that having more cells and having more divisions per cell increases the risk of accumulating a particular set of oncogenic driver mutations. However, the prediction fails when large and small species are compared, since large species do not generally experience an increased cancer risk [25,26]. This apparent contradiction was named ‘Peto's paradox’ and the evolutionary multi-stage model of carcinogenesis (EMMC) was developed to provide a framework for understanding the resolution of this paradox through adaptive evolution [23,27]. The EMMC is based on the premise that as animal species become larger and/or longer-lived, the incidence of some cancers will increase, reducing fitness, and, as a result, natural selection will favour genotypes that restore fitness through enhanced suppression of those cancers. Strong support for this model has been provided by comparative studies in rodents [28]. For example, this work showed that large rodents (e.g. beaver, capybara) have independently evolved telomerase suppression [29], and that the small but long-lived naked mole-rat has evolved a novel mechanism of contact inhibition [30]. Both of these traits are considered to be anti-oncogenic.

However, the evolutionary response of a species to increased cancer risk resulting from increased size (or lifespan) does not generally apply to individual variation within a species. While the EMMC predicts enhanced cancer suppression will be favoured when a species is selected to become larger, this is not the case within a species, because individuals originating from the same gene pool will have the same level of cancer suppression. Thus, given intraspecific body size variation, equation (1.1) is expected to apply to large and small individuals alike, predicting that larger individuals within a species will have a greater cancer risk.

This general pattern has been verified in two species: humans and dogs [31]. Several large-scale human epidemiological studies have shown that taller individuals have a higher overall cancer risk, due to a higher risk across most types of cancer [32–36]. In humans, the multi-stage model provides an accurate quantitative prediction of this increase in cancer incidence per unit height, based on the change in cell number [37]. However, human variation in adult lean body mass (as approximated by height) is relatively modest, rarely being more than twofold in each sex. By contrast, one of the unique features of the domestic dog is the extraordinary range of size it encompasses, spanning a more than 35-fold difference (e.g. a 2.1 kg Chihuahua versus a 77 kg mastiff). Supporting the expected pattern of cancer risk, Fleming *et al.* [18] found a clear effect of breed size on cancer mortality, with larger dog breeds being at a much higher risk of cancer than small breeds. This general pattern has been confirmed in a number of very recent studies [4,38,39], with lifetime cancer mortality showing a clear increase with breed size up to a weight of 30–40 kg, with risk appearing to plateau across heavier breeds [18]. This levelling-off of breed risk appears to be inconsistent with the multi-stage model since, all else being equal, it is expected that cancer risk will increase linearly with size (= *C,* see equation (1.1)). But all else is not equal. It has long been known that large dog breeds are typically much shorter lived than small dog breeds (see [40]). While it appears that larger dog breeds age at a faster rate [41], the casual factors remain unclear [40]. In any event, incorporating this shortened lifespan of large breeds can potentially account for the observed plateau in lifetime cancer risk among the largest breeds [31].

Notwithstanding the general effect of body size, large-scale multi-breed studies show that different types of cancer predominate in different breeds, e.g. [20]. Some of these breed differences in cancer risk may be due to the effects of inbreeding. Inbreeding (and the associated loss of genetic diversity) is a well-known cause of an increased incidence of inherited disease in small isolated populations due to the random loss of alleles and the increase (and possible fixation) of mildly deleterious ones. Dog breeds are analogous to such isolated populations, and inbreeding has probably occurred to varying degrees throughout the long history of dog domestication, and in the relatively recent creation of many newly distinct breeds [19,42,43]. There is some evidence for a link between cancer and low genetic diversity in domestic and wild animals [44], so it is important to consider the possibility that some oncogenic mutations may have become unusually common or fixed in some of the most inbred breeds. Indeed, Kraus *et al.* [4] recently found a weak (non-significant) association between low genetic diversity and high cancer risk across dog breeds.

A high risk of specific cancers may also be created from a trade-off with the targets of artificial selection that are exaggerated to create a given breed. For example, there is a very high incidence of osteosarcoma in many large breeds [45], possibly due to the extended period of bone growth resulting from the selection for large size [46]. To identify and interpret the degree to which a breed suffers from an excess incidence of a specific cancer it is important to establish the expected risk as defined by a fit to the multi-stage model.

To investigate the relative influence of body size, inbreeding and other factors on a breed's risk of cancer, a two-step approach was adopted. First the multi-stage model was fitted to the cancer mortality data across dog breeds to determine if the model was able to account for the observed risk variation across the weight and longevity patterns among dog breeds. A failure of the model to fit the data would cast serious doubt on the adequacy of the multi-stage model, and could suggest a role for other factors. In particular, it has been suggested that the lower mass-specific metabolic rate experienced by larger animals, known as Kleiber's law [47] offsets the expected increase in cancer risk with size [48,49]. On the other hand, a fit to the multi-stage model using overall cancer mortality would undermine the metabolic rate hypothesis. In addition, it would provide a baseline for quantifying any excess risk of specific cancers, which in turn adds power to the search for the genetic changes that predispose some breeds to specific cancers [50]. Second, given a good fit to the multi-stage model, the residual variation in cancer risk of breeds around the best-fitted curve can be tested for potential links between excess risk beyond the expected level and the accumulated inbreeding levels of individual breeds and also to much older groupings (clades) of dog breeds.

More generally, there is recent evidence that inbreeding and/or low genetic diversity increases breed morbidity, measured as non-routine veterinary care [51]; however, the effect of inbreeding on breed lifespan remains ambiguous, with one study [3] finding no effect, while Kraus *et al.* [4] found a significant link between low genetic diversity and reduced lifespan. If this link can be verified, then it raises the possibility of either (i) increased cancer in inbred breeds shortens their lifespan, predicting a positive correlation between cancer mortality and inbreeding (as suggested in [4]) or (ii) a shorter lifespan in inbred breeds reduces lifetime cancer mortality (since cancer is primarily a disease of old age), which predicts a negative correlation.

## 2. Material and methods

The relationship between breed weight and cancer risk was examined using three independent sources of data, all documenting the proportion of deaths in a breed (at any age) due to different causes including cancer. Fleming *et al*. [18] analysed mortality patterns in 81 breeds using US data from the VMDB (US data) with sample sizes per breed ranging from 104 (borzoi) to 4398 (Labrador retriever); Kraus *et al.* [4] analysed data on 119 breeds from the Finnish kennel club (FINN data) with sample sizes of 72 (Tibetan mastiff) to 2333 (Finnish hound); and Adams *et al*. [17] documented mortality in 72 breeds using data from questionnaires to dog owners of pure-bred dogs in the UK (UK data). Four breeds were eliminated from the UK data due to a low number of mortality records (less than 25), leaving samples ranging from 28 (Brittany) to 927 (golden retriever). Together the three studies covered 146 different dog breeds (electronic supplementary material, data). Two combined datasets were also analysed, ALL THREE consisting of the 36 breeds included in all datasets, and ALL MEANS consisting of the 85 breeds with two or three cancer mortality estimates (electronic supplementary material, data). Dachshund was considered three breeds in the FINN data (one standard and two miniature), two breeds in the US data (standard and miniature) and one breed (that combined both standard and miniature forms) in the UK data, so Dachshund was included as a single breed in the ALL THREE dataset, but as two breeds (standard and miniature) in the ALL MEANS dataset, e.g. for the FINN data, the two miniature breeds were averaged for ALL MEANS, and then this value was averaged with the standard breed for ALL THREE. Similarly, the three forms of the Belgian shepherd and two forms of the collie in the FINN dataset were averaged when datasets were combined. To equitably combine the US, UK and FINN data in these pooled datasets, the cancer mortality from each was independently normalized based on the 36 shared breeds so that they each had the original average mean and variance of the three datasets for these breeds. The normalized mean cancer mortality for the ALL MEANS and ALL THREE datasets are listed in the electronic supplementary material, data.

For each breed, the US data documented the proportion of deaths due to various causes including cancer. For the present analysis, these data were modified to exclude accidental (trauma) deaths since the focus was on intrinsic mortality associated with ageing. Trauma deaths were a significant source of mortality (greater than 10% overall) that showed considerable breed variation in occurrence and disproportionately affected young dogs [18]. The proportion of trauma deaths was probably biased upward by the veterinary source of the data (i.e. the VMDB). For the UK and FINN datasets trauma deaths were not excluded since breed-specific values were not provided; however, in the UK data the overall proportion of trauma deaths was known to be low (less than 2.5%).

To determine the fit of the breed data to the multi-stage model, estimates of breed weight and lifespan were needed. Breed weight is a relatively imprecise parameter and large variation was noted across different sources. To obtain a consensus estimate, the following procedure was followed. The average breed weights were based on those used by Fleming *et al.* [18] (unpublished, but with values used provided by the authors) and Adams *et al.* [17] (whose values were obtained from Alderton [52]), with additional data from Kraus *et al.* [41], current American Kennel Club (AKC, https://www.akc.org) values and a set of Australian ‘ideal body weights’ (http://adelaidevet.com.au/pet-library/weight-the-ideal-bodyweight-range-for-your-dog-by-breed). When ranges and/or sex-specific differences were provided, the mean was used (calculated hierarchically, as needed, within sex, then between sexes, then across data source). An overall mean was calculated, noting that not all sources had data for all breeds, and clear outliers relative to the AKC values (greater than 3 s.e. from mean of the non-outliers) were eliminated. This consensus breed weight (electronic supplementary material, data) was used for all analyses except that of the FINN dataset alone when the weights provided in the original report [4] were retained, noting that across the 81 breeds shared between FINN dataset and the US and/or UK datasets the correlation between these breed weights versus the consensus weights was extremely high (Pearson *r* = 0.990).

Unlike breed weight, a breed's lifespan is affected by cancer risk, since a breed with a high level of cancer mortality may have a shortened lifespan. However, the application of equation (1.1) to lifetime cancer risk needs to eliminate the possible effects of high or low cancer risk on the values of lifespan used. This was done by estimating the lifespan for each breed from its weight. Adams *et al*. [17], working with the UK dog population, compared information on median age at death (*T* years) for 81 UK kennel club recognized breeds with their ‘ideal’ body weights (*W* kg) from Alderton [52]. Their regression linking the two variables was *T* = 12.6–0.08 *W*. A second estimate of this relationship was obtained based on weight data and median lifespan data for 77 AKC-recognized breeds in the US that were pooled into seven breed groups (herding, hound, non-sporting, sporting, terrier, toy, working) [53]. Using these data, a linear regression was estimated and compared with the Adams *et al*. [17] regression. If the slope and intercept did not differ between the two datasets, the intent was to combine them into a single regression (weighted by breed number, noting that the US data [53] were derived from multiple breeds pooled into breed groups). The resulting breed estimates of the median age of death are referred to throughout as ‘lifespan’ or ‘expected lifespan’.

Equation (1.1) linking size and lifespan with the incidence of cancer is a simplified form of a more complex relationship. Specifically, equation (1.1) assumes that the genes involved in suppression are tumour suppressor genes, so that knock-out mutations are recessive (i.e. both copies of a gene must be inactivated to eliminate the suppression) and that the somatic mutation rate is constant across the set of driver mutations. Relaxing those assumptions gives

2.1 $$p_{i}=C_{i}T^{M_{i}}\prod_{\;j=1}^{M_{i}}\left(2^{D_{ij}}k_{i}u_{ij}\right),$$

where *D_ij_* defines the recessiveness (= 0) or dominance (= 1) of a mutation *j* driving cancer *i* [23]. Equation (2.1) can be rewritten as

2.2 $$p_{i}=WT^{M_{i}}\left[\;f_{i}\prod_{\;j=1}^{M_{i}}\left(2^{D_{ij}}k_{i}u_{ij}\right)\right]=WT^{M_{i}}F_{i},$$

assuming that the number of vulnerable cells *C_i_* is proportional to overall body weight *W* (i.e. *C_i_* = *f_i_W*) leaving *F_i_* as a constant independent of weight and lifespan (*T*). Further, given the biologically reasonable assumption that any cancer *i* is quite rare, the overall cancer risk $\left(\bar{\;p}\right)$ becomes, to a good approximation, the sum of all *n* possible types of cancer,

2.3 $$\bar{\;p}=\;W\overset{n}{\underset{i=1}{\sum }}(T^{M_{i}}F_{i}).$$

Approximate limits can be placed on the *M_i_*, since in the ideal model they are small integers. The results of estimates both old [22,54,55] and new [56–58] suggest a range estimate of 6 ≥ *M_i_* > 1, so that equation (2.3) can be rewritten for the *k*th dog breed as

2.4 $$\bar{\;p_{k}}=\;W_{k}T_{k}^{2}\overset{n_{2}}{\underset{i=1}{\sum }}F_{i}+W_{k}T_{k}^{3}\overset{n_{3}}{\underset{i=1}{\sum }}F_{i}+W_{k}T_{k}^{4}\overset{n_{4}}{\underset{i=1}{\sum }}F_{i}+W_{k}T_{k}^{5}\overset{n_{5}}{\underset{i=1}{\sum }}F_{i}+W_{k}T_{k}^{6}\overset{n_{5}}{\underset{i=1}{\sum }}F_{i},$$

where *n_x_* is the number of different types of cancer initiated by *x* driver mutations.

From equation (2.4), it follows that if lifespan is a constant across breeds independent of weight (*T_k_* = *T*), then the expected incidence of cancer increases linearly with breed weight (*W_k_*),

2.5 $$\bar{\;p_{k}}=\;A_{0}W_{k}.$$

However, to take account of the observed changes in lifespan with breed size, the lifespan (*T_k_*) of breed *k* was estimated using the linear regression described above, i.e.

2.6 $$T_{k}=a-bW_{k},$$

with *a* and *b* becoming constant parameters used in fitting the multi-stage model.

The cancer mortality data were fitted to the multistage model (equation (2.4)) using a stepwise addition process based on the equation

2.7 $$\bar{\;p_{k}}=\;A_{2}W_{k}T_{k}^{2}+A_{3}W_{k}T_{k}^{3}+A_{4}W_{k}T_{k}^{4}+A_{5}W_{k}T_{k}^{5}+A_{6}W_{k}T_{k}^{6},$$

with the *A_i_* (greater than 0) estimated at each step by multiple regression (Levenberg–Marquardt least squares) with the constant term constrained to zero, as required by the model. The process was initiated with only the lowest power term (the left-most term in equation (2.7)) included. The next higher power was added at each step (moving to the right in equation (2.7)) and the model fit evaluated based on the Akaike information criterion (AIC) using a difference greater than 2 to indicate a significant drop in the fit of a model. Since all of the *A_i_* in equation (2.7) must be positive, at the end of each step any terms with negative or non-significant positive values of *A_i_* were discarded and the next highest power added until the higher power terms were non-significant and only significant positive terms remained. Untested single power versions of equation (2.7) were also evaluated. This stepwise regression was applied to the three independent datasets, to the ALL THREE dataset, and to the ALL MEANS dataset.

To determine if any breed showed a significantly elevated risk of cancer mortality relative to the best-fit model (2.7), the Grubbs test was used [59]. If one significant outlier was detected, the test was repeated after deleting the outlier from the analysis. This process was repeated until all outliers were identified.

To determine if breed inbreeding influenced cancer mortality, the ‘excess cancer risk’ (i.e. the residuals measured as the per cent deviation from expectation based on the best fit to equation (2.7)) were correlated with five independent genomic estimates of inbreeding: inbreeding coefficients estimated from single nucleotide polymorphisms (SNP) and whole genome sequencing (WGS) [60] and from runs of homozygosity (ROH) [3]; reduced genetic diversity using genome wide SNPs (HOM); and reduced diversity in loci from the major histocompatibility complex (MHC) [3]. These different measures were available for various breeds in the US, UK and FINN databases. Homozygosity data from Kraus *et al.* [4] were highly correlated to HOM (Pearson *r* = 0.942, d.f. = 57) and hence not used.

The same inbreeding data were correlated with the ‘excess longevity’, measured as the per cent deviation of the ‘observed’ breed lifespan from the lifespan estimated from the size-based regression (equation (2.6)). The observed lifespan was based on the AKC estimates, with the value used for each breed being the mean of the AKC ranges listed, calculated hierarchically as the mean for each sex, and then the overall mean. The expected lifespan of FINN breeds used body weights provided in the original report [4], while the analysis of the US/UK breeds and of the ALL THREE and ALL MEANS datasets used the consensus weight defined above. To test the hypothesis that a high cancer risk results in a shorter observed (i.e. AKC) lifespan, the correlation of excess longevity and excess cancer risk was calculated.

Parker *et al.* [61] recognized 23 clades of dog breeds, based on genetic relatedness, although they also documented substantial inter-clade genetic mixing. Thus, while dog breeds are by no means equivalent to species in their genetic isolation, it was important to demonstrate that the model fit established using the breed data was not being influenced by the over-representation of particular clades. This possibility was tested by identifying all clades with cancer mortality data for at least three breeds, and using all the available (normalized) data to determine the mean cancer mortality and mean size per clade (electronic supplementary material, data). If the model fit was not being biased by one or more over-represented clades, then the best-fit model should be largely unchanged.

Variation in excess cancer mortality among breed clades was examined by comparing the deviations in cancer mortality from the fitted model of breeds within each clade using ANOVA. The analysis of the US, UK, FINN and ALL MEANS datasets included all clades with at least four breeds represented (electronic supplementary material, data). The ALL THREE dataset had too few breeds with four or more representatives for this analysis (only three clades qualified). Differences between clades were documented using Fisher's least significant difference (LSD), and deviations above or below expectation were examined with a sign test. This ANOVA and all other statistical tests employed ProStat v. 6 (Poly Software International). Associations are defined either by the Pearson correlation coefficient (*r*) or its square, the coefficient of determination (*R*^2^).

## 3. Results

The regression of weight (*W*) on lifespan (*T*) using the breed group data of Greer at al. [53] was *T* = 14.0 − 0.08*W* (*R*^2^ = 0.81, 5 d.f., *p* < 0.01). This breed-group regression equation was almost identical to the relationship of *T* = 12.6 − 0.08*W* (*R*^2^ = 0.40, 79 d.f., *p* < 0.001) published by Adams *et al.* [17] based on the median lifespan of 81 breeds. The similarity of group-based and breed-based regressions indicated that related breeds were not biasing the outcome of the original breed-based analysis. Together they give a pooled regression of *T* = 13.3 − 0.08 W*.* This relationship was used to define each breed's lifespan (*T*) and was used in the multiple regression analysis based on equation (2.7). The robustness of this relationship is further illustrated by the estimation of a very similar relationship (*T* = 10.0 − 0.07 W, *R*^2^ = 0.58, 115 d.f.) based on average lifespan (rather than median) by Kraus *et al.* [4].

The three datasets (US, 81 breeds; UK, 68 breeds; and FINN, 119 breeds), derived from different national databases, provided strong evidence that the degree of cancer mortality of specific dog breeds is quite consistent, since mortality estimates derived from the three independent sources were well correlated. For example, using the 36 breeds in each of the three databases (the ALL THREE dataset), the pairwise correlations in breed cancer mortality were highly significant (US/UK *r* = 0.741; US/FINN *r* = 0.590; UK/FINN *r* = 0.744; d.f. = 34, all *p* < 0.001).

When these three datasets (US, 81 breeds; UK, 68 breeds; and FINN, 119 breeds) were fitted separately to the multi-stage model (equation (2.7)), they produced very similar results linking breed weight and cancer deaths (table 1). The overall best-fit model was *p = A*_4_*WT*^4^, consistent with the hypothesis that many canine cancers are initiated by four mutational events. Comparing the US, UK and FINN datasets, the *M* = 4 model provided the unambiguous best fit in two cases (US and UK) and was not significantly different from the best-fit model (*M* = 5) in the FINN dataset. All other models examined were rejected. Similarly, for the ALL MEANS analysis, which included the 85 breeds present in at least two of the underlying datasets (i.e. those breeds with greater than 1 cancer mortality estimate), the *M* = 4 model unambiguously provided the best fit, and for the ALL THREE analysis, which used the means of the 36 breeds present in all three datasets, the fit of the *M* = 4 model was not significantly different from the best-fit (*M* = 3) model (table 1). Again, all other models examined were rejected.

**Table 1.** The relationship among dog breeds between weight (W) and the proportion of mortality due to cancer. The stepwise fit of equation (2.7) to the US, UK and FINN datasets, and to ALL THREE (using only breeds included in all datasets) and ALL MEANS (using all breeds included in two or three datasets) was evaluated based on the AIC. Parameter values used in defining *T* (see equation (2.7)) were *a* = 13.3, *b* = 0.08 (see text) and *n* = the number of breeds included. Models considered equivalent to the best-fit model (*M* = 4), using the criterion of |ΔAIC| < 2 and having significantly positive parameter estimates, are in italics.

equation (2.7) model	Δ AIC relative to *W*.*T*^4^ model				
	US	UK	FINN	ALL THREE	ALL MEANS
	*n* = 81 breeds	*n* = 68 breeds	*n* = 119 breeds	*n* = 36 breeds	*n* = 85 breeds
*W.T*^2^	26.67	13.20	32.06	5.40	28.81
*W.T*^2^ & *W.T*^3^	4.02^a^	3.17^a^	4.43^a^	0.28^a^	4.45^a^
*W.T*^3^	5.81	3.10	11.02	*−1.48*	8.31
*W.T*^3^ & *W.T*^4^	1.73^b^	1.93^a^	1.07^a^	0.04^b^	1.99^a^
Best fit: *W.T*^4^	*0*	*0*	*0*	*0*	*0*
*W.T*^4^ & *W.T*^5^	2.00^b^	1.90^b^	−0.34^a^	0.75^a^	1.62^b^
*W.T*^5^	6.88	3.05	*−1.68*	6.95	3.45
*W.T*^5^ & *W.T*^6^	4.73^a^	3.41^a^	0.28^a^	2.79^a^	3.58^a^
*W.T*^6^	20.53	10.26	3.53	15.67	14.55
estimate of *A*_4_ (±s.e.)	9.03(±0.29) × 10^−7^	8.15(±0.30) × 10^−7^	6.31(±0.24) × 10^−7^	7.97(±0.33) × 10^−7^	7.84 (±0.26) × 10^−7^
*F* (1,*n*-2)	102.1 (*p* = 1.3 × 10^−22^)	45.8 (*p* = 3.4 × 10^−13^)	88.8 (*p* = 3.5 × 10^−24^)	33.1 (*p* = 1.0 × 10^−8^)	100.8 (*p* = 6.2 × 10^−28^)
correlation	0.656	0.431	0.447	0.563	0.610

^a^One of the estimated coefficients (*A_i_*) was negative.

^b^One or more coefficients (*A_i_*) were not significantly different from zero.

To confirm that the model fit was a general property across dog breeds and not the result of an over-representation of any particular group of closely related breeds, the analysis was repeated using the unweighted means of 16 of the 23 breed clades defined by Parker *et al.* [61] that had sufficient representation. The number of breeds per clade varied from 3 to 14. The clade-based result was very similar to the ALL MEANS result, with the *M* = 4 providing the best fit in both with an almost identical *A*_4_ parameter estimate (7.54 × 10^−7^ versus 7.84 × 10^−7^, respectively) (electronic supplementary material, data).

**Figure 1.**
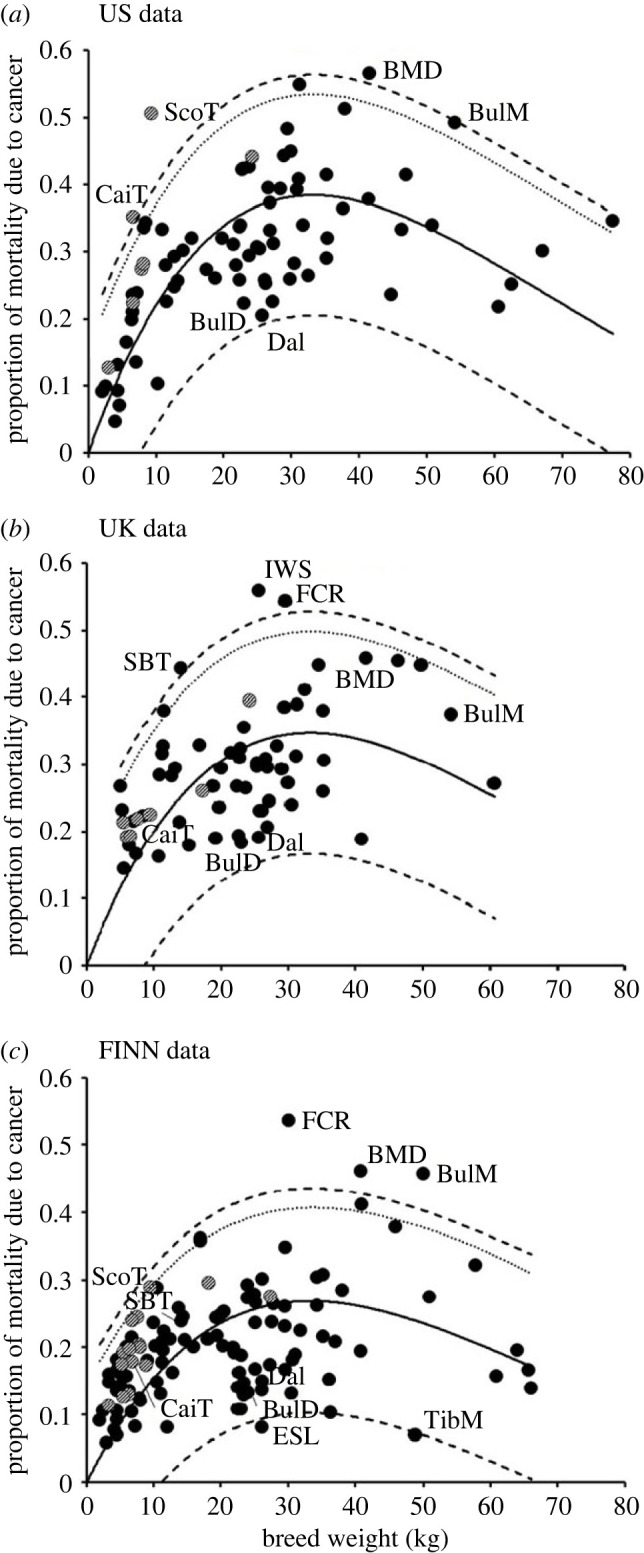
(*a*) US, (*b*) UK, (*c*) FINN dog cancer mortality data plotted with the best-fit *M* = 4 model (solid curve) linking breed weight and cancer mortality (table 1). The dashed lines are the upper and lower 95% prediction bands, and the dotted line is the upper one-tailed 95% prediction band. Two breeds showed significantly elevated cancer mortality: in (*a*) ScoT, Scottish terrier; and in (*c*) FCR, flat-coated retriever. Additional breeds shown with notable high levels of cancer are: BMD, Bernese mountain dog; BulM, bullmastiff; CaiT, Cairn terrier; IWS, Irish water spaniel; and SBT, Staffordshire bull terrier. Breeds shown with notable low levels of cancer are: BulD, bulldog; Dal, Dalmatian; ESL, East Siberian laika; and TibM, Tibetan mastiff. The breeds in the terrier clade (shaded) generally showed higher than expected levels of cancer mortality.

**Figure 2.**
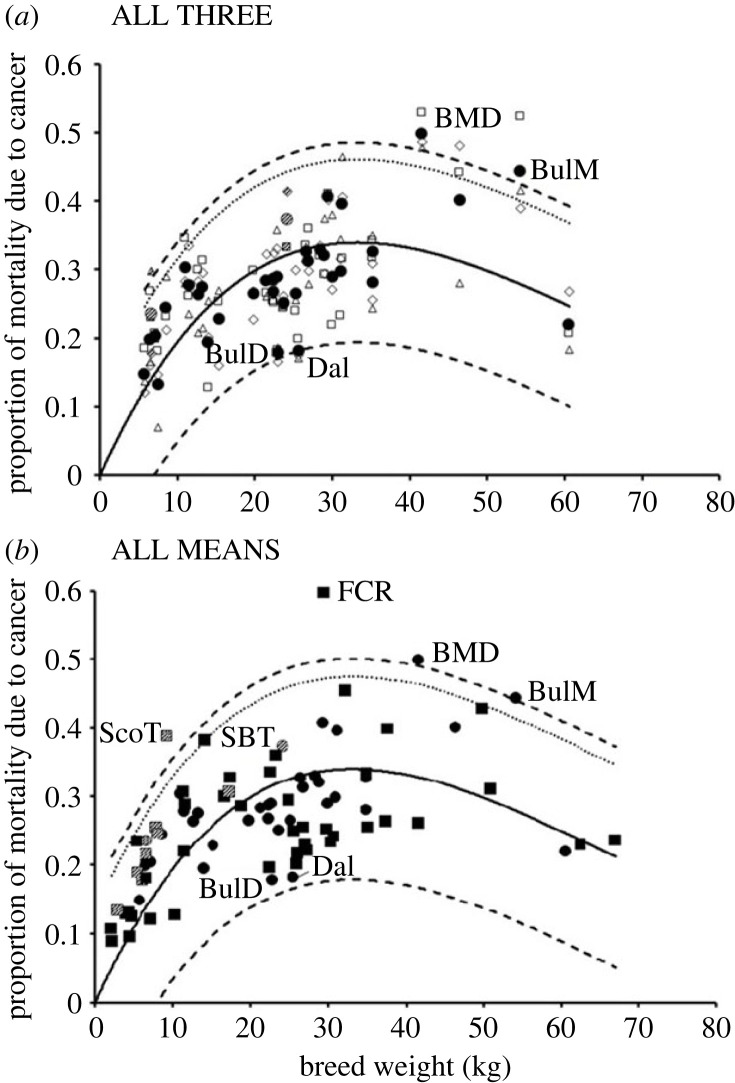
Cancer mortality data for breeds that are (*a*) in all three datasets, and (*b*) in two or three datasets. In both graphs, the best-fit *M* = 4 model (solid curve) linking breed weight and cancer is shown (table 1), together with the upper and lower 95% prediction bands (dashed lines) and the upper one-tailed 95% prediction band (dotted line). The flat-coated retriever, FCR, had a significantly elevated level of cancer mortality, and breeds above the 95% prediction band are also noted: ScoT, Scottish terrier; BMD, Bernese mountain dog; and BulM, bulmastiff. The Staffordshire bull terrier (SBT) is the only additional breed close to significance (*p* < 0.05, one-tailed test). Breeds with consistent (but non-significant) low levels of cancer are: BulD, bulldog; and Dal, Dalmatian. The breeds in the terrier clade (shaded) all show higher than expected levels of cancer mortality. In (*a*) the three values contributing each mean are also shown with open symbols: triangle, US; diamond, UK; and square, FINN. In (*b*) the mean cancer mortality per breed is derived from three datasets (circles) or two datasets (squares).

The *M* = 4 model fit to the five breed-based analyses, combined with the associated prediction bands, was used to determine if any specific breeds had unusually high or low levels of cancer mortality. The three datasets (US, UK FINN) each showed three breeds beyond the upper 95% two-tailed prediction band indicating potentially elevated cancer mortality, involving 7 of the 146 breeds (4.8%) represented across the three datasets (figure 1). Of these breeds only the flat-coated retriever (FINN dataset) and the Scottish terrier (US dataset) were identified as outliers with significantly increased mortality risk (G = 3.26 and 3.34, respectively; both *p* < 0.025). The flat-coated retriever was also in the UK dataset, where it was above the 95% two-tailed prediction band, while the Scottish terrier was also in the FINN dataset, and was above the 90% two-tailed prediction band (figure 1). Of the rest, the Irish water spaniel (UK data) was unreplicated, and two others (Cairn terrier in the US data and Staffordshire bull terrier in the UK data) failed to retain elevated mortality when combined with the other datasets in which they were represented (in UK and FINN; and in FINN respectively; figure 1). The final two, the Bernese mountain dog and the bullmastiff, were more consistent. The Bernese mountain dog was above the 95% two-tailed prediction band in both the US and FINN datasets, while the bullmastiff was above that band in the FINN dataset, and of the 36 breeds in all three datasets, they were the only two whose mean cancer mortality retained evidence of an elevated cancer risk (ALL THREE; figure 2*a*). These two breeds also showed elevated cancer mortality above the 95% prediction band in the ALL MEANS analysis (the 85 breeds present in more than one of the three datasets), together with the flat-coated retriever and the Scottish terrier (figure 2*b*). Thus 4/85 breeds (4.7%) in the ALL MEANS analysis showed evidence of elevated cancer risk, although only in the flat-coated retriever was the risk significantly elevated (G = 3.31; *p* < 0.025).

While a few breeds showed a notable elevation of cancer risk, none showed strong evidence of a reduced cancer risk, although the Dalmatian and bulldog were consistently low (figure 1) and close to the lower 95% prediction band in the combined datasets (figure 2). The East Siberian laika and the Tibetan mastiff also showed some indication of reduced cancer mortality in the FINN data; however, these breeds were only present in one dataset, so this pattern is, as yet, unreplicated.

While the cancer mortality data provided a good fit to the multi-stage model, consistently indicating that cancers requiring *M* = 4 driver mutations dominate, there was substantial unexplained variance. While it is likely that much of this variance is intrinsic to the methods of data collection, another possible reason for variation in cancer mortality is the differences among the breeds in their level of inbreeding. The residual deviations from the best-fit *M* = 4 models (as defined in table 1), expressed as the per cent excess cancer mortality relative to expectation, were correlated with published estimates of inbreeding based on inbreeding coefficients derived from genomic polymorphism (SNP and WGS) and runs of homozygosity (ROH), and on the reverse of heterozygosity estimated from the whole genome (i.e. the lack of heterozygosity; HOM). If inbreeding is linked to breed cancer risk, then a positive correlation is expected. Across the three independent datasets, none of the correlations were significantly different from zero, with 11 of the 12 being negative (table 2). Similarly, the correlations based on the pooled data were also non-significant, with ALL THREE results being positive and the ALL MEANS being negative (table 2). The lack of MHC polymorphism (MHC) was also analysed, but the correlations were small and showed no pattern. In summary, there was no indication that inbred breeds were experiencing a higher proportion of cancer mortality relative to expectation; if anything, the overall pattern suggested the very opposite.

**Table 2.** The correlations among estimates of dog breed inbreeding, excess longevity and excess cancer risk. Five measures of inbreeding across dog breeds were used based on inbreeding coefficients estimated from genetic diversity (SNP and WGS) and from runs of homozygosity (ROH), and from a lack of genetic diversity across the genome (HOM) and at MHC loci. Excess breed longevity was defined by per cent deviation of the observed breed lifespan from expected. Excess cancer mortality was defined similarly by the per cent deviation from the best-fit (M = 4) weight/cancer mortality regression (table 1). The number of breeds in each test (*n*) varied within datasets because the different inbreeding measures covered a different sample of breeds. Significant values are bolded with levels: * *p* < 0.05; ** *p* < 0.01; *** *p* < 0.001.

inbreeding measure	excess breed longevity		excess cancer mortality				
	ALL breeds	FINN breeds	US data	UK data	FINN data	ALL THREE	ALL MEANS
SNP	−0.235* ***p* = 0.042**	−0.341** ***p* = 0.005**	**−**0.206 *p* = 0.131	−0.083 *p* = 0.606	−0.082 *p* = 0.523	0.169 *p* = 0.409	−0.213 *p* = 0.206
	*n* = 75	*n* = 67	*n* = 55	*n* = 40	*n* = 63	*n* = 26	*n* = 37
WGS	−0.331** ***p* = 0.008**	−0.311* ***p* = 0.020**	**−**0.158 *p* = 0.300	−0.044 *p* = 0.808	−0.069 *p* = 0.617	0.035 *p* = 0.880	−0.135 *p* = 0.469
	*n* = 63	*n* = 56	*n* = 45	*n* = 33	*n* = 55	*n* = 21	*n* = 31
HOM	−0.261** ***p* = 0.003**	−0.329*** ***p* = 0.0008**	**−**0.037 *p* = 0.751	0.022 *p* = 0.864	−0.133 *p* = 0.189	0.114 *p* = 0.514	−0.109 *p* = 0.442
	*n* = 124	*n* = 100	*n* = 76	*n* = 63	*n* = 99	*n* = 35	*n* = 52
ROH	−0.108 *p* = 0.295	−0.193 *p* = 0.080	−0.017 *p* = 0.884	−0.162 *p* = 0.266	−0.175 *p* = 0.121	0.178 *p* = 0.306	−0.183 *p* = 0.203
	*n* = 96	*n* = 83	*n* = 76	*n* = 49	*n* = 80	*n* = 35	*n* = 50
MHC	−0.035 *p* = 0.735	−0.062 *p* = 0.573	0.007 *p* = 0.952	−0.040 *p* = 0.760	0.160 *p* = 0.117	0.132 *p* = 0.443	−0.153 *p* = 0.284
	*n* = 96	*n* = 84	*n* = 76	*n* = 61	*n* = 97	*n* = 36	*n* = 51
excess breed longevity	−	−	−0.115 *p* = 0.310 *n* = 80	−0.308* ***p* = 0.011** *n* = 67	0.030 0.758 *n* = 108	−0.327* ***p* = 0.048** *n* = 37	−0.240* ***p* = 0.028** *n* = 84

However, there was a negative relationship between inbreeding and excess breed longevity, defined as the proportional increase in the observed lifespan relative to the lifespan predicted from breed weight. Using all breeds for which inbreeding estimates were available, analysis of the breeds in the FINN dataset (previously examined in [4]) and of all breeds in the combined US/UK/FINN data showed that in both cases all three measures of inbreeding based on genetic diversity were significantly negatively correlated to the excess longevity, i.e. inbred breeds showed a significantly shorter observed lifespan (based on AKC estimates) than was predicted from breed weight. The effect was significant and accounted for 5.5–11% of the variation in excess longevity (table 2). The two inbreeding estimates based on ROH, though negative, were small and non-significant, and there was no indication that MHC diversity influenced lifespan (table 2).

As noted above, to minimize the possibility of the estimates of lifespan used in the fitting of the multi-stage model being affected by variation in cancer mortality among breeds, lifespan was based on the weight–lifespan regression. This approach was validated by the finding of a significant negative correlation between excess longevity (the per cent increase of the observed AKC lifespan over the predicted lifespan) and excess cancer mortality in the ALL MEANS, ALL THREE and UK analyses, with the remaining two correlations (US and FINN) non-significant (table 2).

A second way to look for a potential link between cancer incidence and inbreeding was to consider only the four breeds that showed consistently high cancer mortality (FCR, BMD, BulM, ScoT; figure 2*b*). All four were more inbred than the average breed; however, none were highly inbred, with a range of 0.44–0.88 s.d. above the mean level of inbreeding (averaged across SNP, HOM, WGS, and ROH). For comparison, the estimate of HOM for the grey wolf was 1.4 s.d. above the overall average, which was comparable to those of the four ‘high cancer’ breeds, ranging from 0.63 to 0.74, and 1.88 (Scottish terrier) s.d. above the mean.

Another source of cancer-related mortality could be the effects of inbreeding and/or selection that pre-date the formation of the modern breeds. The clade-based fit of equation (2.7) discussed above was not designed to identify such heterogeneity among the breed clades. To this end, the US, UK, FINN and ALL MEANS datasets were subjected to an ANOVA comparing the excess cancer mortality of all breeds within each clade that had four or more breed representatives (9, 7, 13 and 9 clades per dataset respectively). In all cases, it was found that there were significant differences among the clades (electronic supplementary material, data), with the terrier clade having the highest excess cancer mortality in all four tests. No other clades had a notable excess or deficit of cancer mortality based on *a posteriori* testing.

Across the three independent datasets, all but one terrier breed estimate (1/29) had a greater cancer risk than expectation (figure 1) and this one lower estimate was masked by a second value above expectation from the same breed (soft-coated wheaten terrier), so that the means of all 10 terrier breeds with greater than 1 cancer mortality estimate were above expectation (figure 2). This ALL MEANS result is highly significant (sign test, *p* = 0.002) and the clade averaged a 55% increase above expectation. By contrast, the pointer/setter clade showed some indication of lower-than-expected cancer risk, averaging a 16% drop below expectation in the ALL MEANS dataset, with all eight breed means being below expectation (*p* = 0.008). However, the pointer/setter clade was not consistently the lowest clade group across the three independent datasets (electronic supplementary material, data).

## 4. Discussion

The primary aim of this project was to determine if the multi-stage model of carcinogenesis could account for the patterns of cancer mortality seen across dog breeds. It was found that the quantitative fit to the model (equation (2.7)) was very good (table 1, figures 1 and 2), with a fairly linear increase in cancer mortality with weight in small dogs that plateaus in medium-sized dogs and declines in the largest dogs. The best-fit model was consistent across the three independent datasets, defining a single value of *M* (the number of driver mutations). In terms of the multi-stage model, *M* = 4 suggests that a majority of cancers causing mortality in dogs are initiated by four driver mutations.

An important feature of this fit to the multi-stage model is that the value of *M* = 4 is arguably within a very narrow range of values that would be predicted *a priori*. This expectation can be derived by comparing dogs and humans. The predominant level of cancer suppression (as defined by *M*) in humans is unknown, but, in support of the early estimates [22,54,55], it is still generally considered to encompass the range 2 ≤ M ≤ 7 across different cancer types, e.g. [57,58]. Martincorena *et al.* [56] evaluated multiple cancer types and estimated an average number of positively selected driver mutations in coding regions to be four per tumour. However, regulatory changes are also known to be involved in the initiation of cancer [62], so an average number of about five per tumour is perhaps more accurate.

Given the lower weight (roughly half) and shorter reproductive life (roughly a fifth) of wolves relative to humans, the EMMC predicts a slightly lower average level of cancer suppression in wolves and their descendant dogs than in humans. Specifically, the evolutionary multi-stage model of carcinogenesis (EMMC) predicts that the level of cancer suppression is expected to reduce the fitness loss due to a specific cancer in a given species to approximately 1/(2*Ne*), where *Ne* is the effective population size of the species [23,27]. Assuming that the effective size of ancestral wolf and human populations were not too different, then a similar frequency of fatal cancers is expected during their reproductive lives. Using equation (1.1), this effect can be roughly quantified to determine if the empirical estimate of *M* = 4 driver mutations based on the fit of the multi-stage model leads to a plausible value of *uk* (the product of the somatic mutation rate and the cell division rate). From equation (1.1), the equilibrium cancer mortality (*p*) for the two species is:

4.1 $$p=C_{\mathrm{human}}{\left(ukT_{\mathrm{human}}\right)}^{Mh}=\;C_{\mathrm{wolf}}{\left(ukT_{\mathrm{wolf}}\right)}^{Mw},$$

and using the human and wolf parameters as noted above: *C*_human_ = 2 × *C*_wolf_; *T*_human_ = 5 × *T*_wolf_; *Mh* (human driver mutations) = 5; *Mw* (wolf driver mutations) = 4, combined with a reproductive life of the wolf of about 10 years, then equation (4.1) gives: *uk* = 1.6 × 10^−5^. Further, assuming that the probability of a somatic driver mutation (*u*) is 10^−6^ per division, then the rate of cell division becomes *k* = 16, or one stem cell division per 23 days, a value consistent with estimates for a range of human tissues [63]. This ‘hand waving’ argument illustrates that the fit of the dog cancer mortality data to the multi-stage model with *M* = 4 is consistent with expectation

The fit of the dog breed data to the multi-stage model shows that artificial selection for increased or decreased body size has had, on average, the predicted effect on cancer risk. A similar predictable size effect (based on height) is also seen in humans [37]. By contrast, cancer risk does not change with size across mammalian species [25,26]. Together these patterns unambiguously support the EMMC: increasing body size leads to a greater risk of cancer unless this effect is reduced by a concomitant adaptive increase in cancer suppression [23,27]. As such, the results from dog breeds strongly contradict an alternative non-adaptive hypothesis based on Kleiber's law [47]. Kleiber's law is a well-established relationship linking increased body size to a reduced mass-specific metabolic rate, and, while the precise relationship is debated [64], the negative relationship is consistent, and it has been suggested that this reduction in the cell-level metabolic rate negates the expected increase in oncogenic mutations as body size increases [48,49]. However, Kleiber's law applies within as well as between species. Previously it was found that, based on human data, the metabolic rate hypothesis could not resolve the effect of size on cancer risk, although the possibility of a partial contribution could not be eliminated [65]. The dog data, encompassing a much greater size range, provide a clearer picture. It is known that Kleiber's law applies across dog breeds [66], with the slope of the log-log regression of mass-specific metabolic rate on weight (−0.16) being the same across dog breeds and across canid species [40], and yet there was no indication of an unexplained reduction in the predicted cancer risk with increased body size that could be assigned to the effect of a reduced metabolic rate. Therefore, there is no support for the metabolic rate hypothesis.

As expected, based on the multi-stage model, the smallest breeds in the ALL MEANS dataset have the lowest cancer risk: Chihuahua and Pomeranian were the smallest, and, together with the Pekinese, had the lowest cancer mortality (less than or equal to 10%). However, due to the interaction of size and lifespan, the breeds with the highest levels of cancer mortality were not the largest. The top 3.5% of breeds (3/85) and six of the top nine for cancer risk were in the range 29–42 kg, while the three largest breeds (greater than 60 kg) showed lower levels of cancer mortality (figure 2*b*). All else being equal, the largest breeds are expected to have the highest cancer risk, but since cancer is primarily a disease of old age, the low cancer risk of the very large breeds results from their shortened lifespan.

The fit to the multi-stage model supports the assumption that selective breeding in dogs has not changed the levels of cancer suppression associated with size variation among mammalian species. The absence of breeds that show a significant reduction in cancer mortality relative to the model expectation further supports this view. Dogs are typically chosen for breeding when relatively young, and reducing cancer incidence is not expected to be the target of selection. For example, osteosarcoma is prevalent in some large breeds and by far the leading cause of cancer mortality in the Scottish deerhound, but 80% of cases are diagnosed in animals over age 7 years [67]. It results in about 15–20% of deerhound deaths [68] even though selection to suppress the disease is expected to be successful given that the disease has a substantial heritable component (*h*^2^ = 0.69) [69].

Inbreeding is a well-known cause of an increased incidence of inherited disease in small isolated populations, due to the random increase and possible fixation of mildly deleterious alleles. Dog breeds are somewhat analogous to such isolated populations, so it was important to consider the possibility that some oncogenic mutations may have become unusually common in highly inbred breeds. Kraus *et al.* [4] found a weak (non-significant) positive relationship between cancer mortality and inbreeding (as defined negatively by heterozygosity); however, in the present study there was no evidence of a positive relationship. In all three of the datasets and in the ALL MEANS combined dataset, all of the correlations with SNP, WGS, HOM and ROH were negative but non-significant; only those from the much reduced ALL THREE dataset gave small positive results (table 2). In addition, the four breeds with a significantly elevated cancer risk were not notably inbred; however, the absence of a general effect of inbreeding on cancer mortality does not negate the possibility that some breeds may have experienced an increased cancer risk due to inbreeding.

Mixed breed dogs live about 1.2 years longer than pure bred ones of similar size [3], indicating that a drop in the level of inbreeding is beneficial; however, the same study did not find a link between breed inbreeding and excess longevity. By contrast, a very recent study did find a significant positive correlation between heterozygosity and excess longevity in the breeds in the FINN dataset [4]. The present study supported this last result, first using the same FINN dataset breeds but using three different sources of estimated inbreeding, and second using an enlarged sample of breeds by adding those from the US and UK datasets. In all cases there was a significant negative correlation between excess longevity (i.e. an observed lifespan greater than the expected size-adjusted lifespan) and low genetic diversity (HOM), and a significant negative correlation between excess longevity and two independent estimates of breed inbreeding coefficients using genomic diversity (SNP and WGS) (table 2). An average of 7.7% of the variance around the fitted lifespan/body-size regression could be explained by these three measures of inbreeding (table 2). However, two other measures of inbreeding showed no effect. First, the loss of MHC diversity due to inbreeding has been proposed as an important indicator of the extinction risk faced by species (see [70]); however, there was no link in the dog-breed data of MHC diversity to either increased cancer risk or shortened lifespan (table 2). Second, runs of homozygosity (ROH), which reflect recent inbreeding, also showed no significant correlation with cancer risk or excess longevity. The lack of a significant negative correlation with excess longevity perhaps indicates that the effect of inbreeding on breed lifespan generally pre-dates the relatively short time frame measured by ROH.

Overall, these results suggest that historical inbreeding increases some risk factors that can cause premature death; however, there is no general effect on cancer mortality. Of course, these results do not preclude the effect of inbreeding on specific cancers in specific breeds. Moreover, since cancer is primarily a disease of old age, one possible explanation for the prevalent pattern of (non-significant) negative correlations between cancer mortality and inbreeding (table 2) is that the shortened lifespan of inbred breeds slightly reduced their risk of cancer. It was also found that excess cancer mortality reduces lifespan, as would be expected. Thus, increasing a breed's cancer mortality and/or its inbreeding both reduce excess longevity. Since these two effects are uncorrelated (or possibly weakly negatively correlated; table 2) these are independent effects.

An additional factor examined to account for excess or reduced cancer mortality relative to expectation was the possibility of genetic risk shared within a breed clades due to ancestral effects of inbreeding or selection. Only the terrier group was consistently distinguished with high cancer risk. All of the 10 terrier breeds in the ALL MEANS analysis showed a level of cancer mortality above expectation (figure 2*b*), averaging a 55% excess, which corresponds to an 8.0% increase in lifetime cancer mortality. This increased cancer risk could be due to inbreeding or the artificial selection that occurred during the origin of this clade. By contrast, the pointer/setter group showed a lowered risk, corresponding to a 5.1% drop in lifetime mortality, although the effect was somewhat inconsistent across the three independent datasets (electronic supplementary material, data).

Another potential driver of increased cancer risk is the effect of artificial selection resulting in a correlated response increasing one or more specific cancers. Osteosarcoma appears to be an example of this effect, since unrelated large breeds show the highest incidence. The five breeds identified with the highest incidence (odds ratio greater than 10) in the review of Fenger *et al.* [15] represent four different breed clades as defined in [61]: Great Dane, European mastiff clade; Saint Bernard, Alpine; Irish wolfhound and greyhound, UK rural; and Rottweiler, drover.

The effect of body size complicates the identification of breeds that may have unusually high levels of specific cancers, since mid-sized breeds are likely to be over-represented relative to very small breeds because of their higher overall cancer risk. Fitting the multi-stage model to cancer mortality is designed to provide a solution to this problem; however, the elevated cancer risk of breeds identified by the model fitting has generally been recognized before. For example, a study of Swedish dogs listed the three breeds with the highest risk of cancer mortality as the Bernese mountain dog, the Irish wolfhound, and the flat-coated retriever [71]. Interestingly, the Irish wolfhound, which was included in both the US and FINN datasets, was close to expectation in both; however, the Bernese mountain dog was found to have a consistently high cancer mortality across all three datasets, while the flat-coated retriever was the only breed found to have a significantly elevated risk of cancer mortality. A study following a cohort of flat-coated retrievers found a high incidence of soft tissue sarcoma, particularly histiocytic sarcoma [72].

Model fitting highlighted the elevated cancer risk experienced by one small breed, the Scottish terrier. The elevated risk relative to expectation was significant in the US dataset (figure 1*a*), and was still notably high when combined with the FINN data (figure 2*b*). It is known that this breed has a markedly elevated risk of bladder cancer, some 20-fold higher than mixed breeds [73].

Even without model fitting, it is possible to identify notable breed-related effects. Grüntzig *et al.* [20] normalized their data from the Swiss canine registry by referencing cancer incidence to the generic category of ‘crossbred’ dogs. Their relative risk estimates suggested, for example, a high frequency of adenoma/adenocarcinomas in Yorkshire and West Highland white terriers, and it would be valuable to see if this risk in the two terrier breeds, plus the high risk in the Scottish terrier, can be generalized to account for the terrier clade's generally elevated level of cancer mortality. Another finding of note in the study was the intriguing observation that mammary tumours are more prevalent in small breeds relative to large ones. This finding is of particular interest since it is contrary to the general size-related expectation.

In conclusion, the patterns of cancer mortality across dog breeds are well explained by the multi-stage model of carcinogenesis with very few breeds being unusually cancer prone, and with no indication that increased inbreeding is linked to an increased cancer risk. However, inbreeding was shown to have an effect in shortening lifespan, although reduced MHC diversity alone had no effect on lifespan or on cancer risk. One clade, the terriers, appear to share a higher-than-expected cancer risk, perhaps due to some aspect of the selection imposed on the ancestral breed.

The fit of the dog breed data to the multi-stage model has important implications for the study of cancer suppression across species. They clearly demonstrate that changes in body size and lifespan affect the risk of cancer as predicted by the multi-stage model. They also fail to show any effect on cancer risk resulting from the lowered mass-specific metabolism of larger breeds of dogs, and as such strengthen support for the evolutionary multi-stage model of carcinogenesis (EMMC). The EMMC predicts that selection for larger body size and/or greater longevity will inevitably increase cancer risk unless there is successful selection for increased cancer suppression that reduces the occurrence of the specific cancers that lower fitness, thus resolving Peto's paradox [23,27].

## Data Availability

All data sources are published and fully referenced in the body of the paper. Additional derived data are provided in electronic supplementary material [74].
